# Surgical Anatomy of the Superior Mesenteric Vessels Related to Colon and Pancreatic Surgery: A Systematic Review and Meta-Analysis

**DOI:** 10.1038/s41598-018-22641-x

**Published:** 2018-03-08

**Authors:** Ionut Negoi, Mircea Beuran, Sorin Hostiuc, Ruxandra Irina Negoi, Yosuke Inoue

**Affiliations:** 1 Carol Davila University of Medicine and Pharmacy Bucharest, Bucharest, Romania; 2 Department of General Surgery, Emergency Hospital of Bucharest, Bucharest, Romania; 3 Department of Legal Medicine and Bioethics, National Institute of Legal Medicine Mina Minovici, Bucharest, Romania; 4 Department of Gastrointestinal Surgery, Cancer Institute Hospital, Japanese Foundation for Cancer Research, Tokyo, Japan

## Abstract

The surgeon dissecting the base of the mesenterium, around the superior mesenteric vein (SMV) and artery, is facing a complex tridimensional vascular anatomy and should be aware of the anatomical variants in this area. The aim of this systematic review is to propose a standardized terminology of the superior mesenteric vessels, with impact in colon and pancreatic resections. We conducted a systematic search in PubMed/MEDLINE and Google Scholar databases up to March 2017. Forty-five studies, involving a total of 6090 specimens were included in the present meta-analysis. The pooled prevalence of the ileocolic, right colic and middle colic arteries was 99.8%, 60.1%, and 94.6%, respectively. The superior right colic vein and Henle trunk were present in 73.9%, and 89.7% of specimens, respectively. In conclusion, the infra-pancreatic anatomy of the superior mesenteric vessels is widely variable. We propose the term Henle trunk to be used for any venous confluence between gastric, pancreatic and colic veins, which drains between the inferior border of the pancreas and up to 20 mm downward on the right-anterior aspect of the SMV. The term gastrocolic trunk should not be synonymous, but a subgroup of the Henle trunk, together with to gastropancreatocolic, gastropancreatic, or colopancreatic trunk.

## Introduction

The global burden of colorectal cancer parallels the present human development levels, and by 2030 is expected to increase by 60%, to more than 2.2 million new cases and 1.1 million deaths^1^. For colon cancer patients, the surgical resection represents the mainstay of treatment, with a 5-year relative survival of 89.9% and 71.3% for localized and regional stages, respectively^2^. However, the location of the tumor in the right colon is emerging as a significant negative prognostic factor, with a 20% increased risk of death compared with the cancers arising on the left side^3,4^.

During the latest years, the western concept of complete mesocolic excision with central vascular ligation (CME-CVL)^5^ and the eastern D3 lymphadenectomy^6^ proved their oncological superiority over conventional colonic resections, with lower 5-year local recurrence rate and better overall survival^7^. The surgical safety, better perioperative results and non-inferior long-term oncological outcomes were proved for the laparoscopic CME-CVL^8^ or D3 lymphadenectomy (Supplementary Table I)^9,10^. However, these surgical procedures are technically difficult and associated with more intraoperative organ injuries and severe non-surgical complications^11^.

Understanding the complex tridimensional anatomy of the superior mesenteric vein (SMV) and artery (SMA) is of paramount importance to minimize the iatrogenic injuries during modern radical resections for right colon cancers or surgical resection of tumors located in the uncinate process of the pancreas^12–14^. Standard textbooks of surgery are schematic, often contradictory, and do not offer the required anatomical details for one who embark on refined techniques such as CME-CVL, D3 lymphadenectomy for right colon cancers or pancreatic resections for tumors located in the uncinate process. A comprehensive knowledge of the infra-pancreatic SMV and SMA surgical anatomy is required.

The objective of this systematic review is to propose a standardized terminology of the superior mesenteric vessels, resulted from meta-analysis of the existing evidence, with impact in colon and pancreatic resections.

## Results

### Description of studies

#### Results of the search

**Figure 1:**
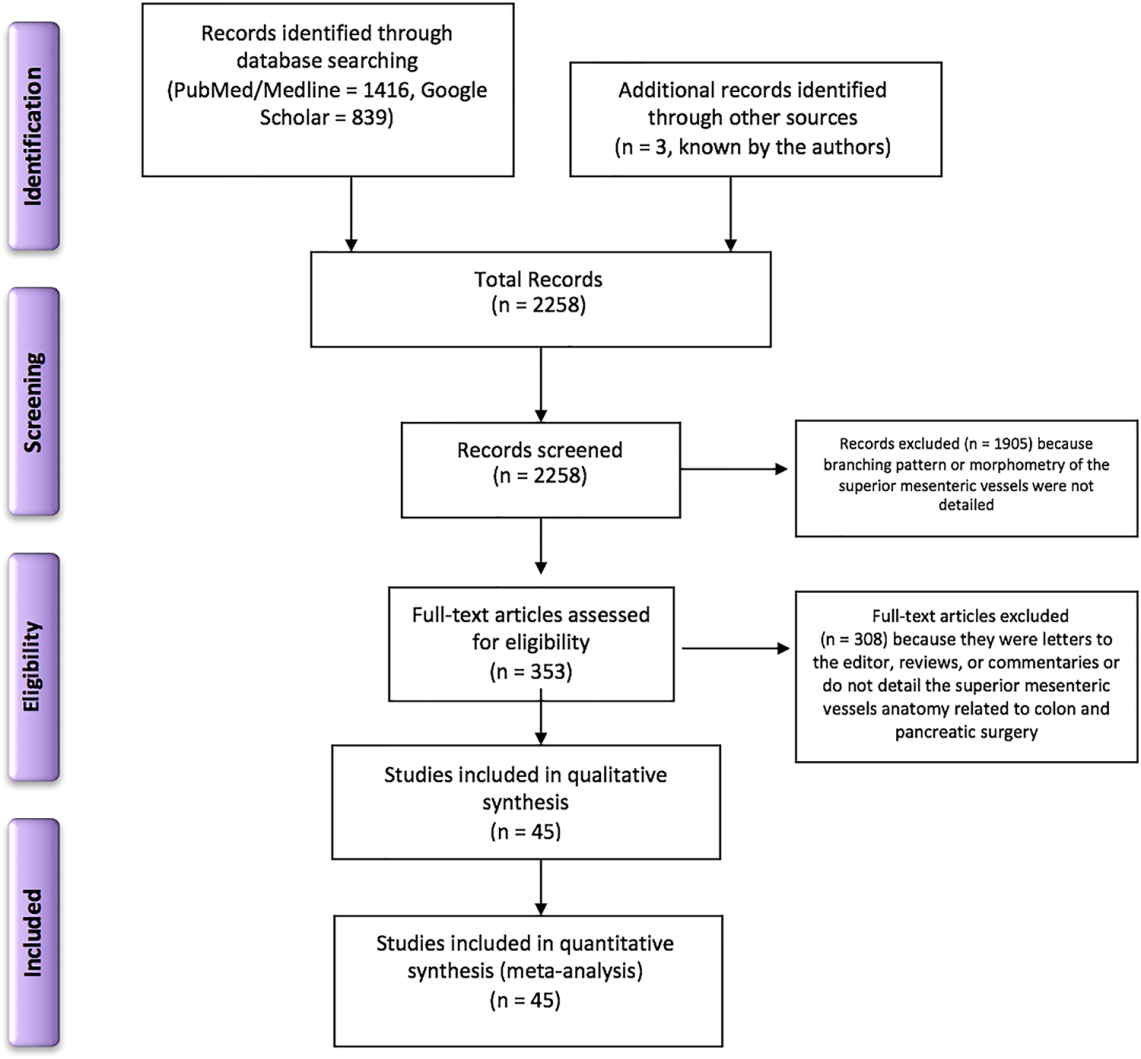
Flow diagram of the systematic literature search and study selection according to PRISMA statement.

The initial electronic and printed literature research retrieved 2258 articles. 1905 papers were excluded after the title and abstract screening, and 353 full-text articles were further evaluated. 308 scientific articles were excluded, and 45 studies, involving a total of 6090 specimens, met the inclusion criteria and were included in the qualitative and quantitative (meta-analysis) synthesis (Fig. 1). 15 studies come from Europe^15–29^, 20 from Asia^30–49^, and ten from the United States of America^50–59^.

#### Included studies

The characteristics of the included studies are summarized in the Table 1. The studies were published between 1909–2017, with a sample size ranging from 9 to 610 specimens. The superior mesenteric vessels and their branches were investigated by dissections of cadaveric specimens in 21 studies^15,16,22–27,29,38,39,44,45,49,52,54–59^, by imaging methods in 16 studies (CT − 13 studies^18,21,28,32–35,37,40,43,50,51,53^, MRI – one study^41^, CT and surgery – one study^20^, Angiography – one study^46^), and by dissection during surgical procedures in eight studies (surgical dissection only – six studies^17,30,31,36,47,48^, surgical dissection and angiography – one study^42^, surgical and cadaveric dissection – one study^19^).

**Table 1:** Characteristics of the included studies.

Reference & Year	Study type	Country	Number of specimens	Main area of interest
Alsabilah 2017	Surgical	South Korea	70	Colon
Kuzu 2017	Cadaveric	Turkey	111	Colon
Murono 2016	Imagistic (CT)	Japan	536	Colon
Gamo 2016	Cadaveric and Imagistic (CT)	Spain	50 + 560	Colon
Haywood 2016	Cadaveric	UK	25	Colon
Lee 2016	Surgical	South Korea	116	Colon
Nesgaard 2015	Imagistic (CT) and Surgical	Norway	139	Colon
Miyazawa 2015	Imagistic (CT)	Japan	100	Pancreas
Kaye 2015	Imagistic (CT)	UK	151	Colon
Cao 2015	Surgical	China	144	Stomach
Ogino 2014	Imagistic (CT)	Japan	81	Colon
Spasojevic 2013	Cadaveric	Norway, Serbia, Switzerland	26	Colon
Hirai 2013	Imagistic (CT)	Japan	100	Colon
Tajima 2011	Surgical	Japan	251	Colon
Spasojevic 2011	Imagistic (CT)	Norway	50	Colon
Sakaguchi 2010	Imagistic (CT)	Japan	102	Gastrointestinal veins
Ignjatovic 2010	Cadaveric	Norway & Serbia& Switzerland	30	Colon
Ignjatovic 2007	Cadaveric	Norway & Serbia	30	Colon
Ferrari 2007	Imagistic (CT)	Italy	60	Abdominal arteries
Jin 2006	Cadaveric	Japan	9	Pancreas + Colon
Cheng 2006	Surgical	China	582	Esophageal replacement
Ignjatovic 2004	Cadaveric	Norway & Serbia	10	Colon
Shatari 2003	Cadaveric	Japan	23	Colon
Yamaguchi 2002	Cadaveric	Japan and USA	58	Colon
Yamada 2000	Imagistic (CT)	Japan	43	Pancreas
Ito 2000	Imagistic (MRI)	Japan	37	Liver
Lange 2000	Surgical and cadaveric	Netherlands	37	Pancreas
Vedantham 1998	Imagistic (CT)	USA	72	Pancreas
Chung 1998	Imagistic (Angiography)	Korea	50	Colon
Yada 1997	Surgery and Angiography	Japan	344	Colon
Graf 1997	Imagistic (CT)	USA	54	Pancreas
Garcia-Ruiz 1996	Cadaveric	USA	56	Colon
Zhang 1994	Cadaveric and Imagistic (CT)	France	110	Gastrocolic trunk
Crabo 1993	Imagistic (CT)	USA	100	Pancreas
Mori 1992	Imagistic (CT)	Japan	66	Gastrocolic trunk
VanDamme 1990	Surgical	Germany	156	Abdominal arteries
Nelson 1988	Cadaveric	USA	50	Abdominal arteries
Birtwisle 1983	Cadaveric	France	50	Anatomy
Michels 1965	Cadaveric	USA	400	Colon
Gillot 1964	Cadaveric + Surgical	USA	81	Colon
Sonneland 1958	Cadaveric	USA	600	Colon
Basmajian 1955	Cadaveric	USA	45	Anatomy
Steward 1933	Cadaveric	USA	50	Colon
Adachi 1928	Cadaveric	Japan	252	Anatomy
Jamieson 1909	Cadaveric	UK	23	Colon

#### Quality assessment of the included studies

The risk of bias according to the authors of the present study was low for 24 studies, moderate for 15 studies, and high for six studies (Supplementary Table II).

The inter-observer agreement was 86.7% (k = 0.779, P < 0.001) for sample representativity for the target population, 93.3% (k = 0.641, P < 0.001) for participants recruitment, 91.1% (k = 0.830, P < 0.001) for the sample size adequacy, 91.1% (k = 0.463, P < 0.001) for the detail of description for subjects and setting, 93.3% (k = 0.536, P < 0.001) for the data analysis, 95.6% (k = 0.727, P < 0.001) for criteria used for measurement of the condition, 95.6% (could not be computed) for the reliability of measurement, 95.6% (k = 0.776, P < 0.001) for statistical analysis, 91.1% (k = 0.831, P < 0.001) for confounding factors and subgroups, and 88.9% (k = 0.845, P < 0.001) for subpopulations identification.

### Pooled prevalence and morphometric data of superior mesenteric artery and vein

#### Ileocolic vessels

**Figure 2:**
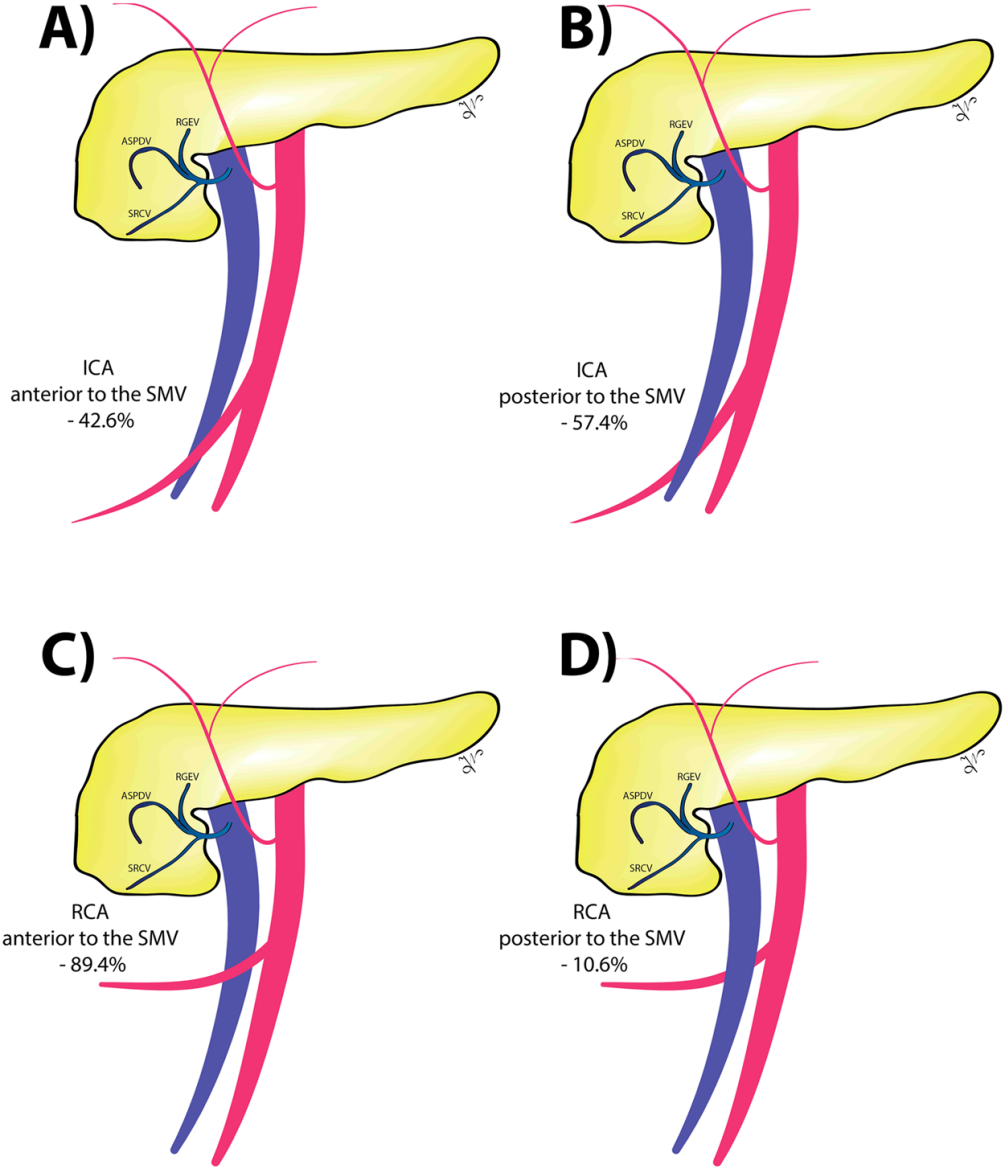
The anatomical relation between the ileocolic (ICA: **A** and **B** images) and right colic (RCA: **C** and **D** images) arteries and the superior mesenteric vein (SMV).

The ileocolic vessels were the most constant anatomical structures, with a pooled prevalence of 99.7% and 99.8% for ilecolic vein (ICV) and artery (ICA), respectively (Table 2 and Fig. 2). The ileocolic vein drainage was into the SMV in 97.6% of cases, into the Henle trunk in 1.9%, and into the jejunal trunk in 0.5% of cases. Related to the SMV, the ICA had a trajectory anterior to the vein in 42.6%, and posterior in 57.4% of cases (Fig. 2).

**Table 2:** Pooled prevalence of the superior mesenteric vein and artery anatomical variants related to colon and pancreatic surgery.

Anatomical parameter		Prevalence	95% CI	I-squared (%)	Cochran’s Q	Chi2, p	Tau2	Luis Furuya-Kanamori (LFK) index
ICV presence		0.997	0.991–1.000	0.000	0.084	0.999	0.000	−3.16
ICV drainage	**SMV**	0.976	0.941–1.000	79.028	14.305	0.003	0.042	0.73
	**Henle trunk**	0.019	0.000–0.056	79.028	14.305	0.003	0.042	−0.90
	**Jejunal trunk**	0.005	0.000–0.028	79.028	14.305	0.003	0.042	1.20
ICA presence		0.998	0.996–0.999	0.000	14.418	0.809	0.000	−3.42
ICA trajectory related to SMV	**Anterior**	0.426	0.363–0.490	80.613	51.581	0.000	0.034	−3.44
	**Posterior**	0.574	0.509–0.636	80.613	51.581	0.000	0.034	3.52
RCV presence		0.591	0.364–0.801	96.774	216.957	0.000	0.378	0.91
RCV drainage	**SMV**	0.490	0.238–0.750	95.608	159.370	0.000	0.489	1.89
	**ICV**	0.008	0.000–0.087	95.608	159.370	0.000	0.489	3.09
	**Henle trunk**	0.503	0.250–0.762	95.608	159.370	0.000	0.489	−1.89
RCVs number	**One**	0.832	0.000–1.000	98.878	267.479	0.000	1.776	0.47
	**Two**	0.134	0.000–1.000	98.878	267.479	0.000	1.776	−0.68
	**Three**	0.034	0.000–0.576	98.878	267.479	0.000	1.776	0.52
SRCV presence		0.739	0.382–0.985	98.078	260.159	0.000	0.656	−2.01
SRCV drainage	**SMV**	0.036	0.000–0.146	93.415	75.927	0.000	0.293	1.14
	**RCV**	0.008	0.000–0.075	93.415	75.927	0.000	0.293	4.38
	**MCV**	0.015	0.000–0.096	93.415	75.927	0.000	0.293	3.54
	**Henle trunk**	0.941	0.726–1.000	93.415	75.927	0.000	0.293	0.86
RCA presence		0.601	0.454–0.741	98.894	2350.586	0.000	0.579	−3.05
RCA origin	**SMA**	0.708	0.337–0.595	97.991	895.830	0.000	0.315	0.49
	**ICA**	0.138	0.028–0.181	97.991	895.830	0.000	0.315	−1.62
	**MCA**	0.154	0.034–0.194	97.991	895.830	0.000	0.315	−1.86
RCA trajectory related to SMV	**Anterior**	0.894	0.856–0.921	5.344	9.508	0.392	0.002	−1.84
	**Posterior**	0.106	0.076–0.141	5.344	9.508	0.392	0.002	1.75
ICA and RCA, with different origins in the SMA, trajectory related to SMV	**ICA anterior & RCA anterior**	0.160	0.089–0.240	84.521	19.381	0.000	0.034	−0.29
	**ICA anterior & RCA posterior**	0.006	0.000–0.028	84.521	19.381	0.000	0.034	2.04
	**ICA anterior & RCA absent**	0.342	0.243–0.438	84.521	19.381	0.000	0.034	−4.70
	**ICA posterior & RCA anterior**	0.109	0.051–0.180	84.521	19.381	0.000	0.034	−1.62
	**ICA posterior& RCA posterior**	0.049	0.012–0.103	84.521	19.381	0.000	0.034	3.28
	**ICA posterior & RCA absent**	0.334	0.235–0.429	84.521	19.381	0.000	0.034	4.24
MCV presence		0.967	0.899–1.000	91.660	95.927	0.000	0.144	−0.57
MCVs number	**One**	0.697	0.514–0.858	93.032	71.760	0.000	0.189	1.88
	**Two**	0.259	0.111–0.441	93.032	71.760	0.000	0.189	−2.40
	**Three**	0.044	0.000–0.137	93.032	71.760	0.000	0.189	0.34
MCV drainage	**SMV**	0.832	0.746–0.896	80.303	30.461	0.000	0.053	−1.12
	**Henle trunk**	0.117	0.059–0.188	80.303	30.461	0.000	0.053	−0.37
	**SV**	0.015	0.000–0.044	80.303	30.461	0.000	0.053	−0.03
	**IMV**	0.019	0.000–0.050	80.303	30.461	0.000	0.053	3.01
	**FJT**	0.018	0.000–0.048	80.303	30.461	0.000	0.053	2.20
MCA presence		0.946	0.902–0.979	90.758	151.490	0.000	0.092	0.08
MCAs number	**One**	0.884	0.819–0.945	63.919	5.543	0.063	0.019	−0.93
	**Two**	0.106	0.053–0.177	63.919	5.543	0.063	0.019	1.17
	**Three**	0.010	0.000–0.034	63.919	5.543	0.063	0.019	−1.51
MCA origin	**SMA**	0.787	0.374–0.968	98.344	181.147	0.000	0.411	−1.34
	**RCA**	0.178	0.000–0.442	98.344	181.147	0.000	0.411	2.50
	**HA**	0.006	0.000–0.111	98.344	181.147	0.000	0.411	4.24
	**SA**	0.003	0.000–0.093	98.344	181.147	0.000	0.411	5.42
	**LCA**	0.008	0.000–0.121	98.344	181.147	0.000	0.411	4.09
	**ICA**	0.008	0.000–0.121	98.344	181.147	0.000	0.411	4.09
	**Celiac artery**	0.003	0.000–0.093	98.344	181.147	0.000	0.411	5.42
	**IPDA**	0.006	0.000–0.111	98.344	181.147	0.000	0.411	4.24
Henle trunk presence (RGEV + CVs+/−PVs)		0.897	0.830–0.948	91.393	209.126	0.000	0.158	−0.35
Henle trunk types	**GCT**	0.045	0.000–0.203	98.153	433.235	0.000	0.857	2.57
	**GPT**	0.337	0.029–0.594	98.153	433.235	0.000	0.857	1.48
	**GPCT**	0.605	0.165–0.796	98.153	433.235	0.000	0.857	−0.43
	**CPT**	0.013	0.000–0.127	98.153	433.235	0.000	0.857	2.16
Henle trunk forming veins	**GCT (RGEV** **+** **SRCV)**	0.054	0.000–0.149	97.975	592.632	0.000	0.671	3.27
	**GPT (RGEV** **+** **ASPDV)**	0.267	0.032–0.395	97.975	592.632	0.000	0.671	0.72
	**CPT (ASPDV** **+** **SRCV)**	0.011	0.000–0.074	97.975	592.632	0.000	0.671	2.09
	**GPCT (RGEV** **+** **ASPDV** **+** **1 Colic: RCV)**	0.059	0.000–0.155	97.975	592.632	0.000	0.671	1.92
	**GPCT (RGEV** **+** **ASPDV** **+** **1 Colic: SRCV)**	0.386	0.080–0.490	97.975	592.632	0.000	0.671	−0.80
	**GPCT (RGEV** **+** **ASPDV** **+** **1 Colic: MCV)**	0.021	0.000–0.096	97.975	592.632	0.000	0.671	2.57
	**GPCT (RGEV** **+** **ASPDV** **+** **1 Colic: ICV)**	0.009	0.000–0.071	97.975	592.632	0.000	0.671	3.40
	**GPCT (RGEV** **+** **ASPDV** **+** **2 Colic: RCV** **+** **SRCV)**	0.095	0.000–0.201	97.975	592.632	0.000	0.671	−0.12
	**GPCT (RGEV** **+** **ASPDV** **+** **2 Colic: RCV** **+** **MCV)**	0.026	0.000–0.105	97.975	592.632	0.000	0.671	1.74
	**GPCT (RGEV** **+** **ASPDV** **+** **2 Colic: SRCV** **+** **MCV)**	0.023	0.000–0.100	97.975	592.632	0.000	0.671	2.15
	**GPCT (RGEV** **+** **ASPDV** **+** **2 Colic: RCV** **+** **ICV)**	0.010	0.000–0.072	97.975	592.632	0.000	0.671	2.36
	**GPCT (RGEV** **+** **ASPDV** **+** **3 Colic: RCV** **+** **SRCV** **+** **MCV)**	0.027	0.000–0.107	97.975	592.632	0.000	0.671	2.45
	**GPCT (RGEV** **+** **ASPDV** **+** **3 Colic: RCV** **+** **SRCV** **+** **ICV)**	0.012	0.000–0.076	97.975	592.632	0.000	0.671	2.84
Henle trunk drainage	**SMV trunk**	0.816	0.149–1.000	95.154	20.636	0.000	0.768	—
	**Right intestinal trunk of the SMV**	0.184	0.000–0.851	95.154	20.636	0.000	0.768	—
Anatomical parameter		**Mean**	**95% CI**	**I-squared (%)**	**Cochran’s Q**	**Chi2, p**		**Tau2**
ICA crossing length (mm)		15.151	13.902–16.400	40.6	3.367	<0.001		0.506
ICA to RCA distance (mm)		15.973	13.889–18.057	0	0.441	<0.001		0.000
RCA crossing length (mm)		20.686	18.531–22.842	37.6	3.209	<0.001		1.381
Henle trunk distance from inferior border of the pancreas (mm)		7.459	−2.121–18.039	97.3	37.814	0.167		56.778
Henle trunk diameter (mm)		3.9	3.083–4.720	98.9	645.092	<0.001		1.375
Henle trunk length (mm)		14.202	11.639–16.764	79.8	9.889	<0.001		4.044

CI – confidence interval; CPT – colo-pancreatic trunk; GCT – gastro-colic trunk; GPT – gastro-pancreatic trunk; GPCT gastro-pancreato-colic trunk; HA – hepatic artery; ICA – ileocolic artery; ICV – ileocolic vein, IMV – inferior mesenteric vein; IPDA – inferior pancreaticoduodenal artery; FJT – first jejunal trunk; LCA – left colic artery; MCA – middle colic artery; MCV – middle colic vein; ASPDV – anterosuperior pancreaticoduodenal vein; RGEV – right gastroepiploic vein; RCA – right colic artery; RCV – right colic vein; SMA – superior mesenteric artery; SMV – superior mesenteric vein; SV – splenic vein; SA – splenic artery; SRCV – superior right colic vein; LFK index within 1 was interpreted as no asymmetry, exceeding 1 but within 2 as minor asymmetry, and exceeding 2 as major asymmetry.

The subgroup analysis of studies with more than 100 included specimens, the continent of origin (Europe, Asia, and the USA), and the method of vessel characterization (imagistic, surgical, and cadaveric) revealed no significant changes in the size of the effects (Supplementary Table III).

The pooled ICA crossing length was 15.2 mm.

#### Right colic vessels

The right colic vein (RCV) was present in 59.1% of cases (Table 2). The RCV’s drainage was into the Henle trunk, SMV, and ICV in 50.3%, 49.0%, and 0.8% of specimens. In 83.2% there was a single MCV, while in 13.4%, and 3.4% there were two, and three MCVs.

The right colic artery (RCA) was present in 60.1% of cases. The origin of the RCA was into the SMA, MCA, and ICA in 70.8%, 15.4%, and 13.8%. The trajectory of the RCA related to the SMV was anterior in 89.4%, and posterior in 10.6% of cases (Fig. 2).

The pooled ICA to RCA distance was 16.0 mm. The mean RCA crossing length was 20.7 mm.

#### Superior right colic vein (SRCV)

The SRCV was present in 73.9% of specimens (Table 2). The SRCV drained into the Henle trunk, SMV, MCV, and RCV in 94.1%, 3.6%, 1.5%, and 0.8% of cases, respectively.

#### Middle colic vessels

The middle colic vein (MCV) was present in 96.7% of cases (Table 2). There was one, two or three MCVs in 69.7%, 25.9%, and 4.4% of specimens. The MCV drained into the SMV in 83.2%, into the Henle trunk in 11.7%, into the inferior mesenteric vein in 1.9%, into the first jejunal trunk in 1.8%, or into the splenic vein in 1.5% of cases.

The middle colic artery (MCA) was present in 94.6% of cases. There was one MCA in 88.4% of cases, two in 10.6%, and three in 1.0%. The MCA origin was in the SMA in 78.7%, in the RCA in 17.8%, in the ICA in 0.8%, in the left colic artery in 0.8%, in the inferior pancreaticoduodenal artery in 0.6%, in the hepatic artery in 0.6%, in the splenic artery in 0.3%, and in the celiac artery in 0.3% of cases.

#### Henle trunk

**Figure 3:**
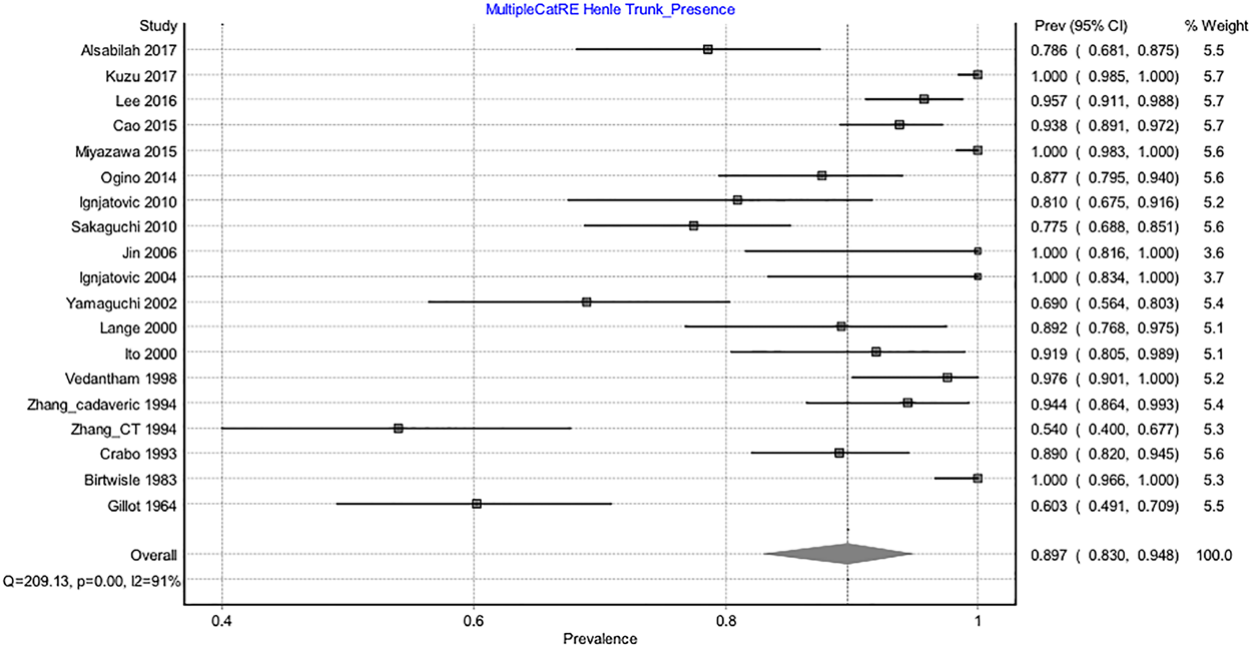
Forrest plot presenting pooled prevalence of the Henle trunk presence.

**Figure 4:**
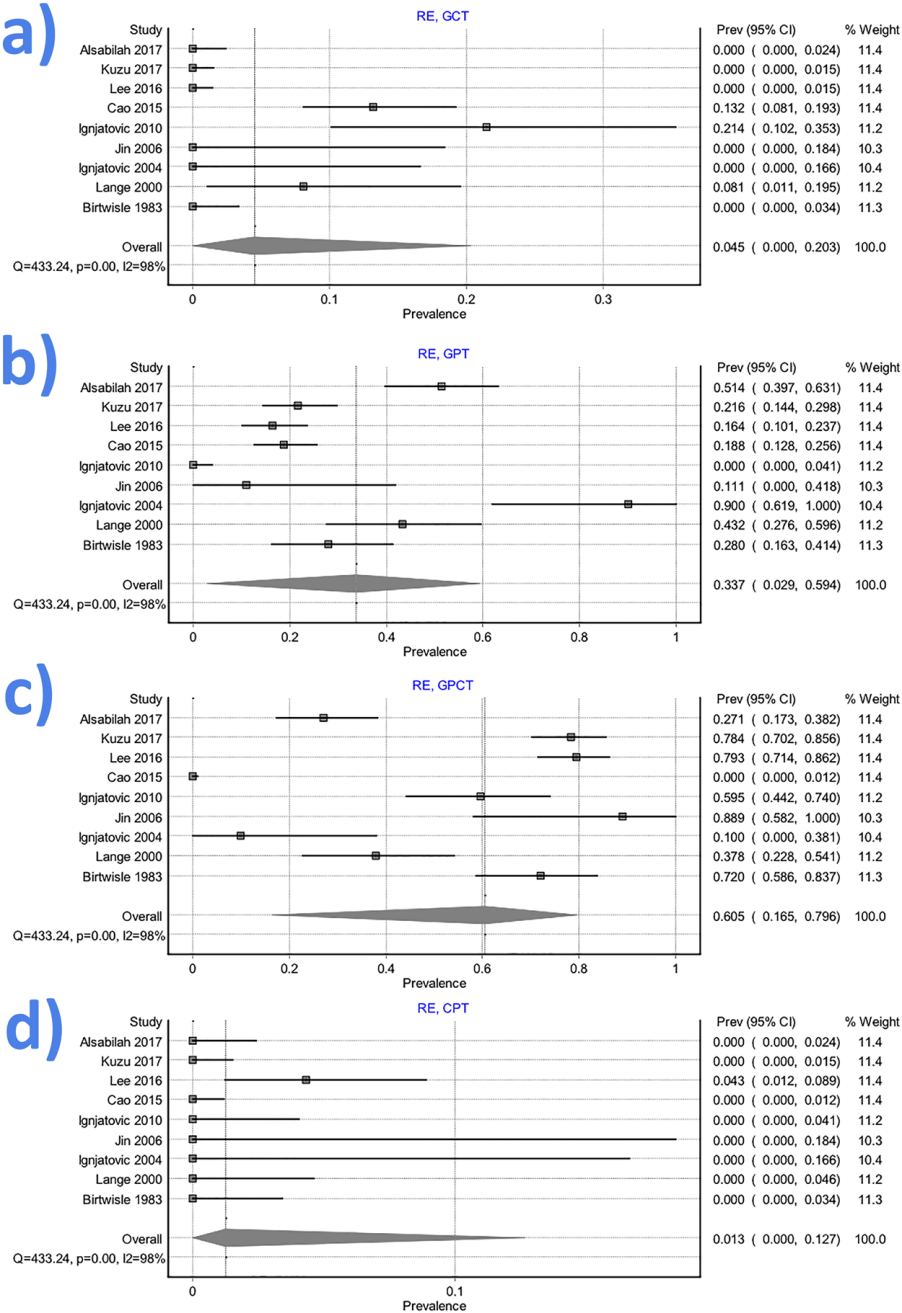
Forrest plot presenting pooled prevalence of the Henle trunk conformations: (**a)** gastro-colic trunk (GCT); (**b**) gastro-pancreatic trunk (GPT); (**c**) gastro-pancreato-colic trunk (GPCT); (**d**) colo-pancreatic trunk (CPT).

The Henle trunk, defined as confluence of the right gastroepiploic vein (RGEV) with one or more colic veins, and with or without a pancreatic vein, was present in 89.7% of specimens (Table 2 and Fig. 3). The Henle trunk was a gastro-pancreato-colic trunk (GPCT) in 60.5%, a gastro-pancreatic trunk (GPT) in 33.7%, a gastro-colic trunk (GCT) in 4.5%, and a colo-pancreatic trunk (CPT) in 1.3% of cases (Fig. 4).

The Henle trunk was a GCT made by the RGEV and SRCV in 5.4% of cases. It was a GPT made RGEV and anterosuperior pancreaticoduodenal vein (ASPDV) in 26.7%, and a CPT made by the ASPDV and SRCV in 1.1% of specimens.

The pooled prevalence of the Henle trunk as a GPCT trunk formed by RGEV, ASPDV and one colic vein was: 38.6% for RGEV + ASPDV + SRCV, 5.9% for RGEV + ASPDV + RCV, 2.1% for RGEV + ASPDV + MCV, and 0.9% RGEV + ASPDV + ICV.

The prevalence of the Henle trunk as a GPCT trunk formed by RGEV, ASPDV and 2 colic veins was: 9.5% for RGEV + ASPDV + RCV + SRCV, 2.6% for RGEV + ASPDV + RCV + MCV, 2.3% for RGEV + ASPDV + SRCV + MCV, 1.0% for RGEV + ASPDV + RCV + ICV.

The pooled prevalence of the Henle trunk as a GPCT trunk formed by RGEV, ASPDV and 3 colic veins was: 2.7% for RGEV + ASPDV + RCV + SRCV + MCV, and 1.2% for RGEV + ASPDV + RCV + SRCV + ICV.

The Henle trunk drained into the SMV, and right intestinal trunk of the SMV in 81.6% and 18.4% of cases, respectively.

**Figure 5:**
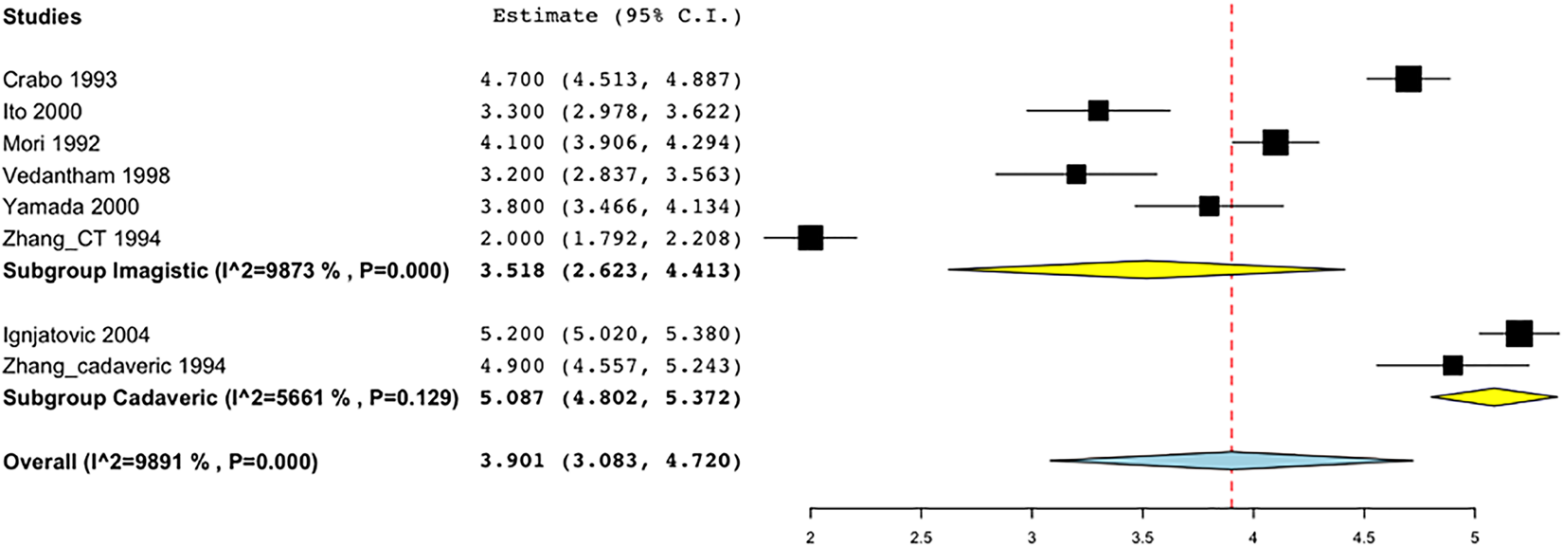
Forrest plot presenting pooled mean value with subgroup analysis (imagistic versus cadaveric) for Henle trunk diameter.

The Henle trunk had a pooled mean diameter of 3.9 mm (Fig. 5), and a mean length of 14.2 mm. The pooled mean distance between the inferior border of the pancreas and the emergence of the Henle trunk was 7.5 mm.

### Publication bias

Sensitivity analysis was conducted to assess statistical heterogeneity, through the exclusion of specific studies with high risk of bias (Supplementary Table IV). There were no relevant changes in the overall effects of the quantitative synthesis. Analysis of the LFK index revealed no asymmetries for 19 outcomes, minor asymmetries for 17 outcomes, and major asymmetries for 33 outcomes (Table 2, Supplementary Figures 1 and 2).

## Discussions

The present systematic review and meta-analysis demonstrates anatomical variants of the superior mesenteric vessels with impact in surgical dissection during radical resections for right colon and pancreatic head cancer. The superiority of meta-analyzing the anatomical findings over simply pooling the results is that data of individual studies are weighted initially, then combined^60^.

Over the latest decade, the implementation of minimally invasive surgery has dramatically increased in the field of colorectal surgery, given is proven superior perioperative outocomes^61,62^. However, ongoing concerns were regarding the quality of the resected specimen and the long-term oncological outcomes, especially for the most refined techniques such as CME-CVL or D3 lymphadenectomy during right hemicolectomy^63,64^. The right hemicolectomy with CME-CVL has a long, and without a plateau learning curve, correlating with the complex anatomy and necessity for meticulous dissection around critical structures^65^. The reported conversion rate in laparoscopic colectomy is 10–20%, one of the most frequent reasons for that being bleeding^66,67^.

**Figure 6:**
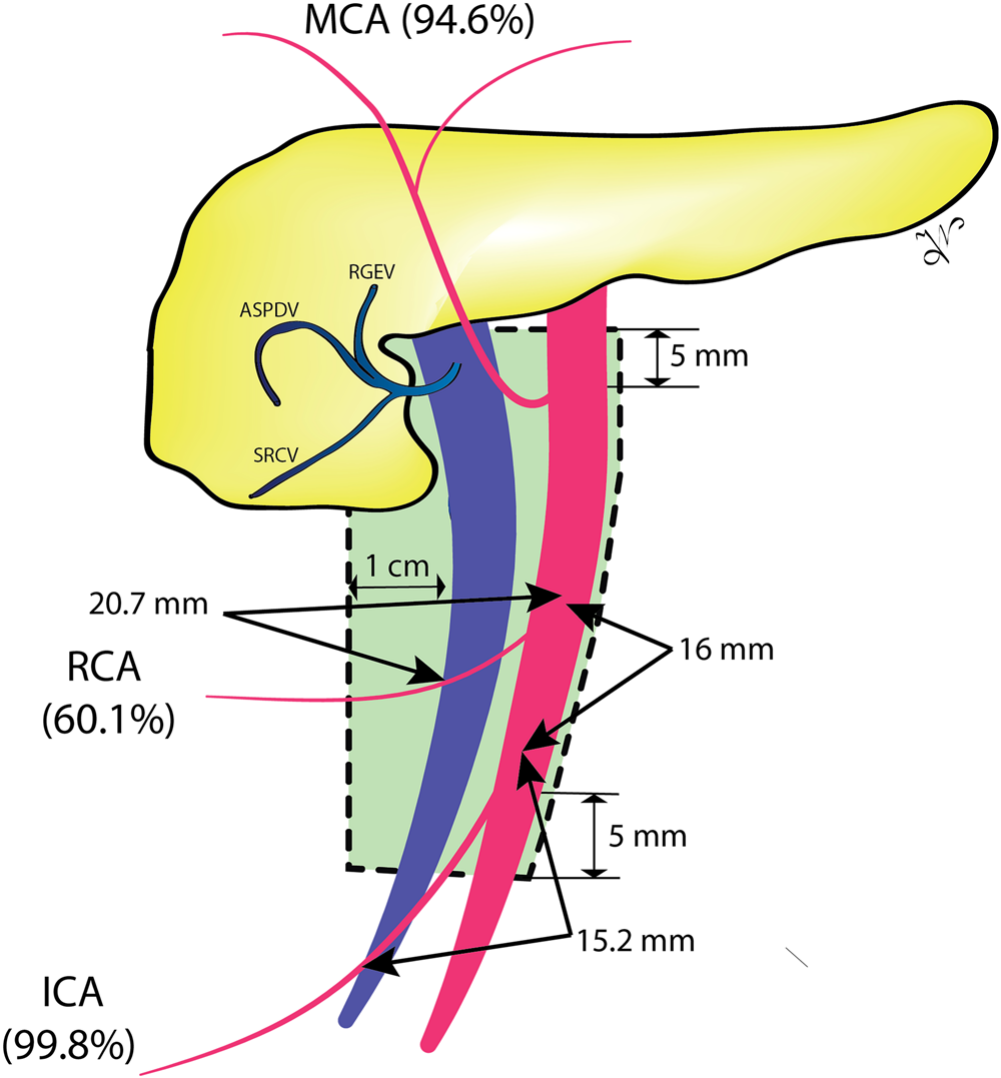
The boundaries of the D3 area (green area) and the frequency of presence for the ileocolic artery (ICA), right colic artery (RCA), and middle colic artery (MCA). It can be observed the ICA and RCA crossing lengths, and the pooled distance between the ICA to RCA origin distance.

The CME-CVL or D3 lymphadenectomy require ligation of the ICV, RCV, Henle trunk, and MCV on their emergence from the SMV, and of the ICA, RCA, and MCA on their emergence from the SMA. In our meta-analysis, we found a wide range of anatomical variability of the major vascular structures, which suggests that surgical dissection during right hemicolectomy with CME-CVL is not straightforward, and should be done carefully, following the embryological planes. The D3 area has the following anatomical boundaries: (a) cranially – five mm proximal to the horizontal line through the Henle trunk and MCA origins; (b) caudally – five mm distal to the horizontal line through the origin of the ICA; (c) medially – the left edge of the SMA; (d) laterally – one cm from the right edge of the SMV^25^. Should be noted the difficulty of the CME-CVL surgical technique, which requires reflection of SMV to centrally ligate the colic arteries^68^. A recent concept included the ICA and RCA crossing lengths, which are the length of these arteries which traverse the anterior or posterior aspects of the SMV^22^. We found a pooled mean ICA, and RCA crossing lengths of 15.2 mm, and 20.7 mm, respectively (Fig. 6). The reported incidence rate of metastasis in central ileocolic lymph nodes was up to 11.1%, which justifies the surgeon struggling to centrally ligate the vessels^42,52,69^.

In the present study, the ICA and ICV were the most constant anatomical structures and should be used as landmarks for starting dissection along the SMV axis. The RCA and RCV were the most inconstant anatomical structures. The middle colic vessels were constantly present. Should be noted that right and middle colic arteries were also multiple, two or even three, in a significant number of cases. The reported rate of intraoperative bleeding during minimally invasive colectomies range from 3% to 9.2%^70^. We found that ICA and RCA had a trajectory posterior to the SMV in 57.4% and 10.6%, which suggests the high risk of vein injury when the operating surgeon try to control bleeding from one of these pedicles, retracted posteriorly to the SMV.

The SRCV was a common anatomical structure in our study, being present in almost 74% of specimens. Should be noted the anatomical difference between the RCV, which drains the blood from the marginal veins of the ascending colon and the SRCV which drains the hepatic flexure of the colon. We consider inappropriate the terminology of accessory RCV or MCV be used for the anatomical structure that drains the hepatic flexure of the colon. We propose a common terminology which should include the SRCV terminology.

**Figure 7:**
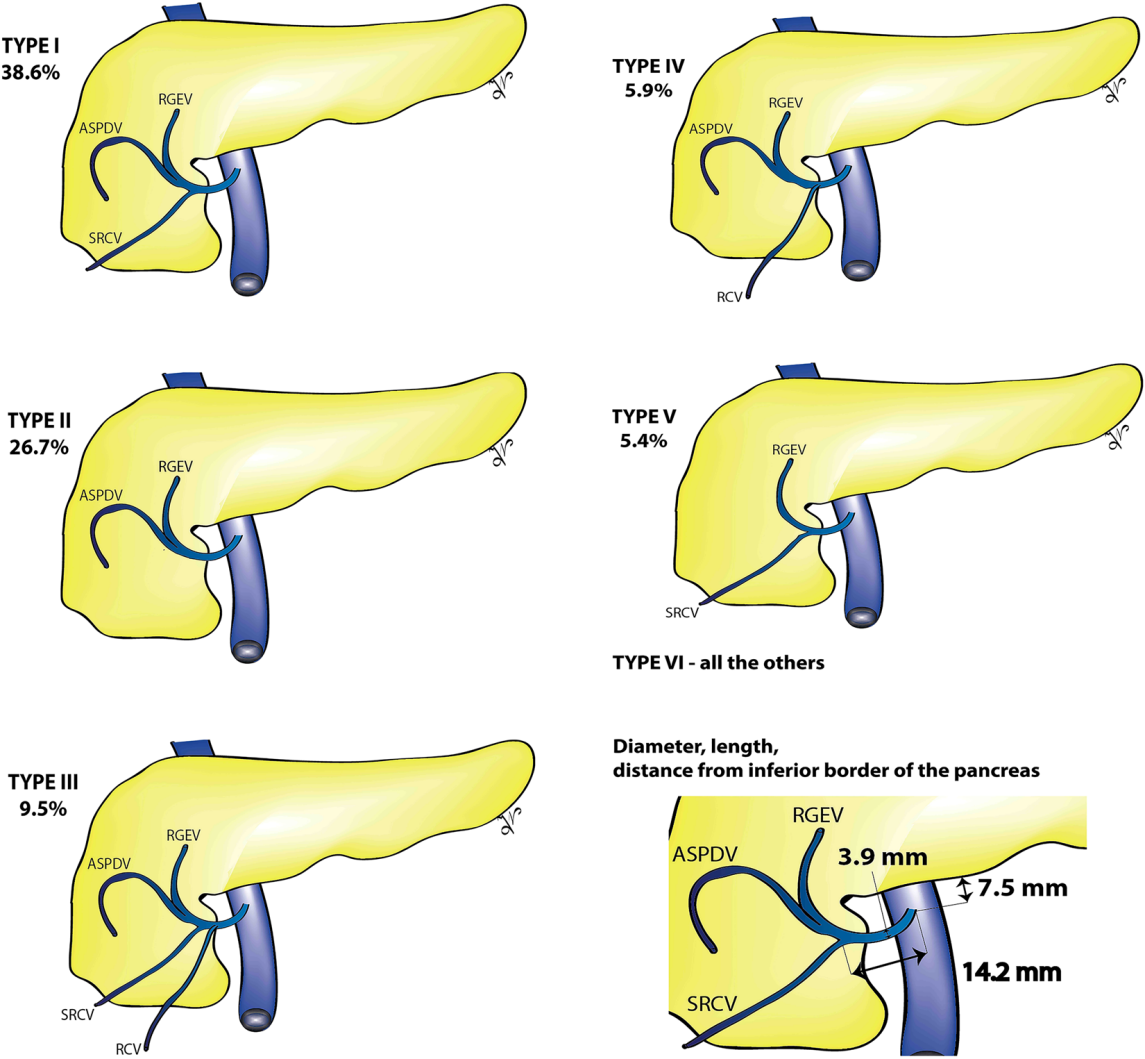
Our proposed standardized terminology for Henle trunk surgical anatomy. ASPDV – anterosuperior pancreaticoduodenal vein; RGEV – right gastroepiploic vein; RCV – right colic vein; SRCV – superior right colic vein. To proposed a common terminology for Henle trunk, we grouped all the anatomical variants with a pooled prevalence less than 5.0% in the ‘other’ group of ‘Type VI’. Should be noted that Type I has the highest pooled prevalence, and the Type V the lowest.

The Henle trunk had a very complex and highly variable tridimensional anatomical structure. In 1868, Henle described a venous confluence formed by the RGEV and the superior right colic vein^71^, and Descomps and De Lalaubie added in 1912 the third element, the ASPDV^56,72^. We are proposing a standardized terminology, with impact in the right colon, pancreatic, and gastric oncological resections (Table 3 and Fig. 7). We propose the term Henle trunk to be used for any venous confluence between gastric, pancreatic and colic veins, which drains between the inferior border of the pancreas and up to 20 mm downward on the right-anterior aspect of the SMV. We propose that term ‘gastrocolic trunk’ should not be synonymous, but a subgroup of the Henle trunk, together with to ‘gastropancreatocolic, gastropancreatic, or colopancreatic trunk’. To propose a common terminology, easy to be implemented in clinical practice, we grouped all the anatomical variants with a pooled prevalence less than 5.0% in the ‘Type VI’ (Table 3). The Type I has the highest pool prevalence, and the Type V the lowest, but higher than 5%. Usually, intraoperative bleeding occurs through inadvertent traction by the surgical assistant, with tearing of these fragile veins.

**Table 3:** Proposed terminology for Henle trunk based of the pooled prevalence resulted from the present meta-analysis.

Proposed terminology	Veins which confluence to form the Henle trunk	Pooled prevalence	Anatomical groups		
**Type I**	RGEV + ASPDV + SRCV	38.6%	RGEV + ASPDV + 1 colic vein	GPCT	Henle trunk
**Type II**	RGEV + ASPDV	26.7%		GPT	
**Type III**	RGEV + ASPDV + RCV + SRCV	9.5%	RGEV + ASPDV + 2 colic veins	GPCT	
**Type IV**	RGEV + ASPDV + RCV	5.9%	RGEV + ASPDV + 1 colic vein	GPCT	
**Type V**	RGEV + SRCV	5.4%		GCT	
**Type VI (all others)**	RGEV + ASPDV + RCV + SRCV + MCV	2.7%	RGEV + ASPDV + 3 colic veins	GPCT	
	RGEV + ASPDV + RCV + MCV	2.6%	RGEV + ASPDV + 2 colic veins	GPCT	
	RGEV + ASPDV + SRCV + MCV	2.3%	RGEV + ASPDV + 2 colic veins	GPCT	
	RGEV + ASPDV + MCV	2.1%	RGEV + ASPDV + 1 colic vein	GPCT	
	RGEV + ASPDV + RCV + SRCV + ICV	1.2%	RGEV + ASPDV + 3 colic veins	GPCT	
	ASPDV + SRCV	1.1%		CPT	
	RGEV + ASPDV + RCV + ICV	1.0%	RGEV + ASPDV + 2 colic veins	GPCT	
	RGEV + ASPDV + ICV	0.9%	RGEV + ASPDV + 1 colic vein	GPCT	

CPT – colo-pancreatic trunk; GCT – gastro-colic trunk; GPT – gastro-pancreatic trunk; GPCT gastro-pancreato-colic trunk; ICV – ileocolic vein, MCV – middle colic vein; ASPDV – anterosuperior pancreaticoduodenal vein; RGEV – right gastroepiploic vein; RCV – right colic vein; SRCV – superior right colic vein. To proposed a common terminology for Henle trunk, we grouped all the anatomical variants with a pooled prevalence less than 5.0% in the ‘other’ group of ‘Type VI’. Should be noted that Type I has the highest pooled prevalence, and the Type V the lowest.

Bertelsen *et al*. showed that CME-CVL technique is associated with higher rate of intraoperative organ injuries (9.1% vs. 3.6%, P < 0.001), including SMV lesions (1.7% vs. 0.2%, P < 0.001)^11^. The CME-CVL group had a higher rate of sepsis requiring vasopressors (6.6% vs. 3.2%, P = 0.001) and respiratory failure (8.1% vs. 3.4%, P < 0.001)^11^. Freund *et al*. described five cases (1.6%) of SMV injuries from a total of 304 radical right colectomies^73^. Only two of these injuries were observed during the initial surgery, and three patients required saphenous graft reconstruction, with one postoperative death^73^.

Preoperative planning of the right hemicolectomy and pancreatic resection, based on high-quality imaging, is expecting to decrease the rate of adverse intraoperative events while improving the quality of the resected specimen^33,74,75^. Mari *et al*. showed that patient’s vascular mapping using CT angiography, before right hemicolectomy (38 patients), significantly reduced the operating time (130 16.3 vs. 147 28.2 minutes, P = 0.027), decreased the difficult identification of the mesenteric vessels intraoperative identification of the SMV (1 vs. 7 cases, P = 0.053), and decreased the intraoperative bleeding (P = 0.006)^76^.

Laparoscopic pancreaticoduodenectomy is a very complex procedure, which expands its indications and clinical implementation worldwide. The current evidence proposed a hospital threshold of 22 cases per year to minimize the associated postoperative complications^77^. However, in experienced centers, the long-term oncological outcomes of minimally invasive approach are non-inferior to the open surgery^78^.

The uncinate process pancreatic cancers (UPPC) have been regarded as tumors associated with an ominous prognosis and even lower resection rate compared with similar tumors located in the pancreatic head^14^. This is attributed mainly to their very intimate relationships with the superior mesenteric vessels^79^. A study comparing 161 patients with UPPC with 292 non-UPPC patients showed that uncinate tumors had a higher rate of SMA invasion (P < 0.001), lower resectability (P = 0.003), and lower R0 resection rate (22.3% vs. 35.6%, P = 0.003)^80^. After R0 resection, the UPPC patients had a poorer overall survival (median 21 vs. 26 months, P = 0.018), with a higher local recurrence rate (P = 0.038) and early occurrence of the local relapse (median 13 vs. 52 months, P < 0.001)^81^. We consider that careful preoperative planning of the surgical technique, and understanding of the complex vascular anatomy from the base of the mesenterium is especially important in patients with tumors located in the uncinate process of the pancreas. Miyazawa *et al*. used the tridimensional CT to map the Henle trunk vascular anatomy in 120 patients before pancreaticoduodenectomy^33^. The authors concluded that understanding of the vascular anatomy might prevent bleeding in the separation of the pancreas and transverse colon during pancreaticoduodenectomy, especially in obese patients^33^. For invasive pancreatic cancers in the uncinate process, venous resection including spleno-mesenteric junction is often required to achieve R0 resection. In such cases, a marginal vein in the hepatic flexure later becomes a thick collateral drainage of the splenic venous flow^82^. SRCV often forms a part of this marginal way, and should be ligated as central as possible to preserve the passway. Careless sacrifice of SRCV at peripheral part causes defect of marginal passway, leading to intraluminal varices at the hepatic flexure or bleeding of varicose veins. If preservation of SRCV nor right colic vessels were not possible due to cancer invasion, concomitant right colectomy is needed. In such a case, reconstruction of the splenic vein would be an option to prevent postoperative sinistral portal hypertension.

Although the most comprehensive study in the literature about the topic according to our knowledge, should be acknowledged that this meta-analysis has several limitations, especially due to the heterogeneity of the terminology used in the included studies. Another important limitation of the current study is related to the inherent differences between imagistic, surgical and imagistic methods of vessel characterization. Third, between the included studies there was a significant variability of the patients’ geographical origin, number of specimens, and pre-existing morbidities. However, by using the random effects model for pooled data and a large number of specimen analyzed we minimized the effects of heterogeneity.

## Conclusions

The infra-pancreatic anatomy of the superior mesenteric vessels is widely variable. The surgical dissection during right hemicolectomy with CME-CVL is not straightforward and should be done carefully, following the embryological planes. We propose the term Henle trunk to be used for any venous confluence between gastric, pancreatic and colic veins, which drains between the inferior border of the pancreas and up to 20 mm downward on the right-anterior aspect of the SMV. The term gastrocolic trunk should not be synonymous, but a subgroup of the Henle trunk, together with to gastropancreatocolic, gastropancreatic, or colopancreatic trunk.

## Methods

We followed the Preferred Reporting Items for Systematic Reviews and Meta-Analyses (PRISMA)^83^ and Meta-Analysis of Observational Studies in Epidemiology (MOOSE)^84^ guidelines in conducting and reporting the results of this systematic review and meta-analysis.

### Data sources and search strategy

We searched the PubMed/MEDLINE and Google Scholar databases, up to March 31, 2017. The search strategy combined key words related to the superior mesenteric vein and artery surgical anatomy. We used no language restrictions. We screened the reference list of the full-text articles to identify additional relevant studies. The search strategy used in PubMed/Medline database was detailed in Supplementary Table V.

### Study selection

Study eligibility criteria: We included all the studies detailing the branching pattern and morphometric data of the SMV and SMA. Exclusion criteria: (1) conference proceedings; (2) sample in a specific subset of the general population (e.g. portal hypertension patients); (3) animal studies; (4) case reports, review articles, editorials and letters to the editor; (5) overlapping or duplicate reports.

### Outcome measures

Primary outcomes: branching pattern of the SMV and SMA. Secondary outcomes: anatomical relationships between the venous and arterial branches, anatomical relationships between arterial branches and SMV, morphometric data of the blood vessels with impact in right colon and pancreatic surgical oncology. The clinical questions to be addressed are: (a) Which is an adequate nomenclature for the Henle trunk; (b) Which type of dissection is recommended during right colectomy according to vascular variability; (c) How should central vascular ligation during right hemicolectomy be performed; (d) What is “risky” anatomy of the gastrocolic trunk or mesenteric-portal venous systems during right colectomy or pancreatiododenectomy; (e) Which are the surgical technique options to manage pancreatic tumor located in the uncinate process.

### Data extraction

Data from individual studies were extracted independently by two authors (IN, SH). We used a predefined electronic protocol; the disagreements being resolved by discussion. We extracted from full texts and supplemental materials the following data: year of publication, first author, title, journal, contact address, country of the study, inclusion and exclusion criteria, sample size, demographic data, subgroup of patients, method of vessels investigation, branching pattern of the SMV and SMA, diameter of vessels, anatomical relationships between the venous and arterial branches, distance between the origins of these vessels.

### Quality assessment

We used the JBI Critical Appraisal Checklist for Studies Reporting Prevalence Data^85^ to assess the methodological quality of the included studies. This grades sample representativity for the target population, participants recruitment in an appropriate way, if the sample size is adequate, the detail of description for subjects and setting, if the data analysis was conducted with sufficient coverage of the identified sample, if objective, standard criteria were used for measurement of the condition, the reliability of measurement, if statistical analysis was appropriate, if all confounding factors and subgroups were identified and accounted for, and if subpopulations were identified using objective criteria^85^. For each of the ten domains, we have attributed 2 points for Yes, 1 point for Unclear, and 0 points for No. According to the total score, studies were considered to present a low, moderate or high risk of bias if this was 17, 13–16, 12 points, respectively. Two authors (IN, SH) independently performed the quality assessment. The inter-observer agreement of the quality assessment was calculated using percent agreement and Cohen’s kappa coefficient^86,87^. The disagreements were resolved by a consensus process.

### Statistical analysis

For statistical analysis, we used as statistical software the MetaXL version 5.3 (EpiGear International Pty Ltd, Queensland, Australia)^88^, and openMeta[Analyst]TM^89^ version 12.11.14. The venous and arterial branching pattern was defined by calculating the multi-categorical pooled prevalence. When the estimate for a specific study tends toward 0% or 100%, the variance moves toward zero, and in consequence, its weight is overestimated in a meta-analysis of prevalence^88^. Therefore, we preferred to use the double arcsine transformation over the logit when calculated multiple category prevalences, as this stabilizes the variance and makes it dependent only on the population size^88^. For the continuous data, we calculated the pooled mean of the superior mesenteric vein, artery, or of their branches. We used Cochran’s Q test (^2^) and I^2^ statistics to evaluate the studies’ heterogeneity^90^. The P < 0.1 and a 50% were considered the cut-off value between low and high heterogeneity^91^. To allow the between-study variation, we used the random-effect model meta-analyses^85^. To assess the publication bias we used the Begg’s funnel plot^92^, Doi plot, and Luis Furuya-Kanamori (LFK) index^93^. An LFK index within 1 was interpreted as no asymmetry, exceeding 1 but within 2 as minor asymmetry, and exceeding 2 as major asymmetry. The subgroup analysis and meta-regression considered the influence on the size of the effect of the method of vessels characterization (surgical, imagistic Computed Tomography, Magnetic Resonance Imaging, Angiography or cadaveric dissection or corrosion casts), the continent origin of the study, year of publication, the number of included patients. Reasons for statistical heterogeneity were explored using sensitivity analyses, through the exclusion of specific studies one by one and compared the results.

### Data availability

All the data are available at the corresponding authors and can be offered on request.

## Electronic supplementary material

Supplementary material
