# Supplementary material for: Surgical Anatomy of the Superior Mesenteric Vessels Related to Colon and Pancreatic Surgery: A Systematic Review and Meta-Analysis
Source: Sci Rep. 2018 Mar 8;8:4184. doi: 10.1038/s41598-018-22641-x (PMC5843657; doi:10.1038/s41598-018-22641-x)
Supplement: Supplementary file 1 — Supplementary material [file 41598_2018_22641_MOESM1_ESM.pdf]

Surgical Anatomy of the Superior Mesenteric Vessels Related to Colon and Pancreatic Surgery: A Systematic Review and Meta-Analysis

Ionut Negoi[1,2], Mircea Beuran[1,2], Sorin Hostiuc[1,3], Ruxandra Irina Negoi[1], Yosuke Inoue[4]

[1]*Carol Davila* University of Medicine and Pharmacy Bucharest, Romania

[2]Department of General Surgery, Emergency Hospital of Bucharest, Romania

[3]Department of Legal Medicine and Bioethics, National Institute of Legal Medicine *Mina Minovici,* Romania

[4]Department of Gastrointestinal Surgery, Cancer Institute Hospital, Japanese Foundation for Cancer Research, Tokyo, Japan

Supplementary Figure 1: Begg's funnel plot for the pooled prevalence of the Henle trunk presence.

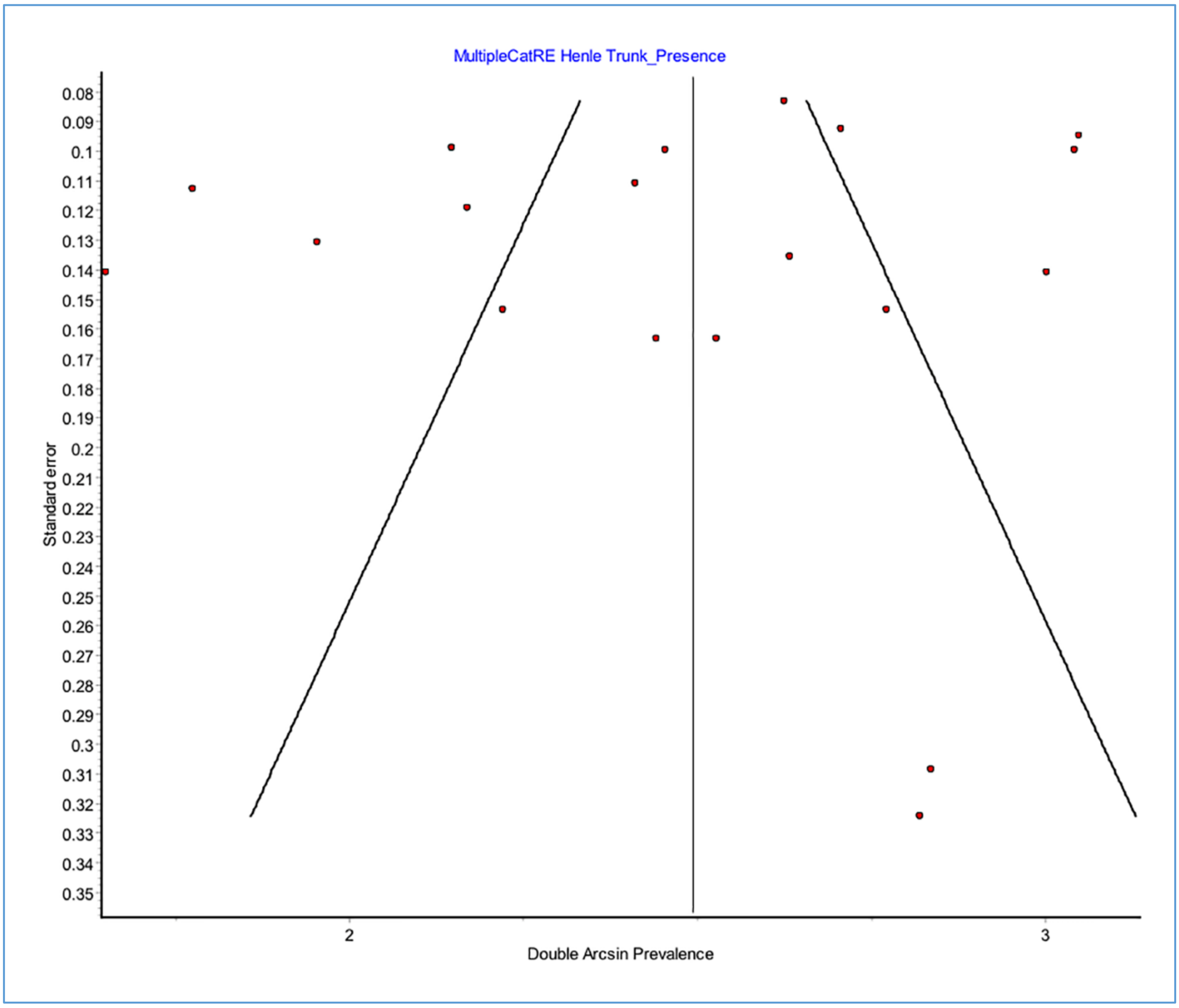

Supplementary Figure 2: Doi plot for the pooled prevalence of the Henle trunk presence.

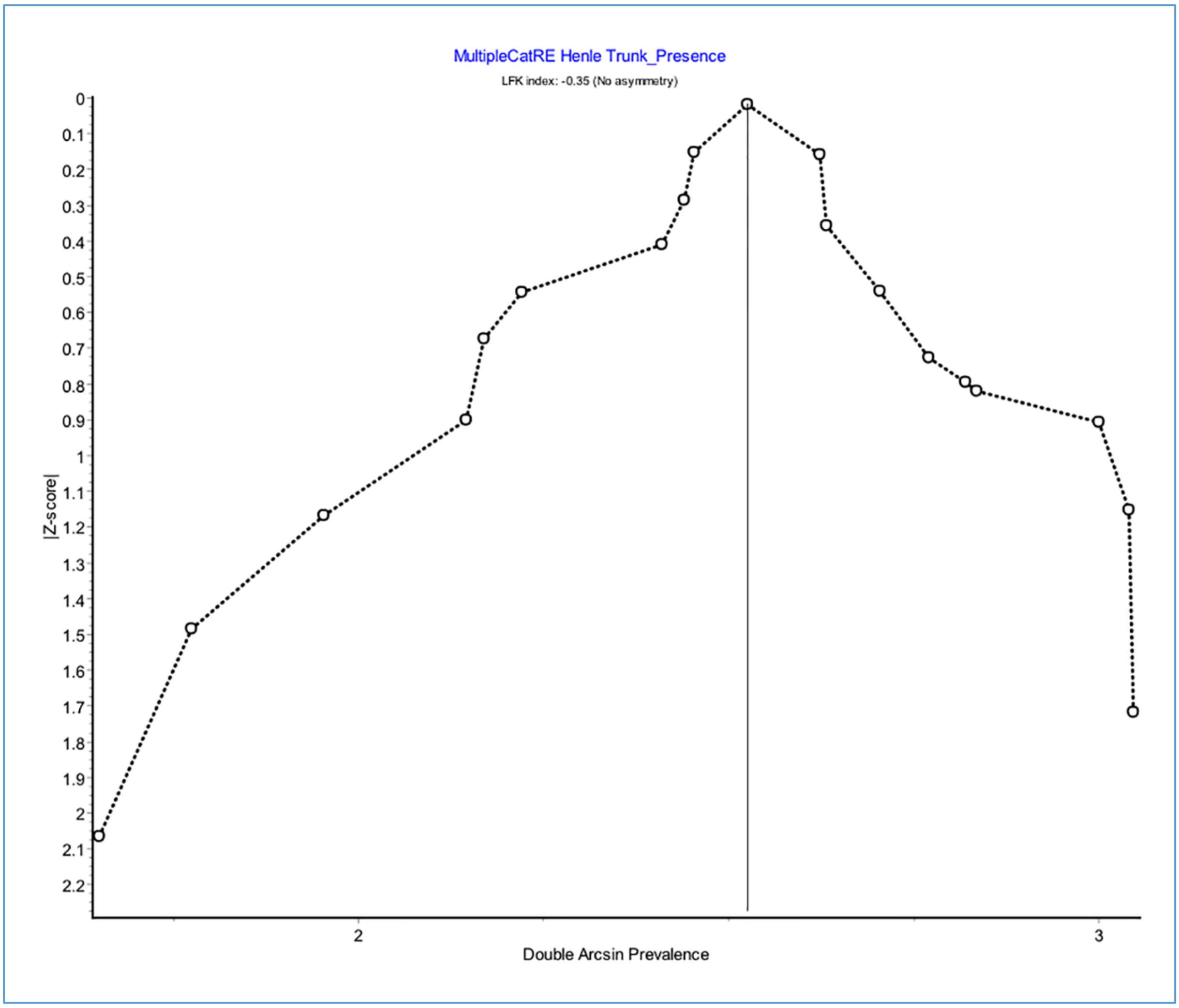

Supplementary Table I: List of abbreviations.

ASPDV	Anterosuperior Pancreaticoduodenal Vein
CME-CVL	Complete Mesocolic Excision with Central Vascular Ligation
CPT	Colo-Pancreatic Trunk
CT	Computed Tomography
GCT	Gastro-Colic Trunk
GPT	Gastro-Pancreatic Trunk
GPCT	Gastro-Pancreato-Colic Trunk
HA	Hepatic Artery
ICA	Ileocolic Artery
ICV	Ileocolic Vein
IMV	Inferior Mesenteric Vein
IPDA	Inferior Pancreaticoduodenal Artery
LFK INDEX	Luis Furuya-Kanamori Index
LCA	Left Colic Artery
FJT	First Jejunal Trunk
MCA	Middle Colic Artery
MCV	Middle Colic Vein
MDCT	Multidetector-Row Computed Tomography
MRI	Magnetic Resonance Imaging
RGEV	Right Gastroepiploic Vein
RCA	Right Colic Artery
RCV	Right Colic Vein
SMA	Superior Mesenteric Artery
SMV	Superior Mesenteric Vein
SV	Splenic Vein
SA	Splenic Artery
SRCV	Superior Right Colic Vein
UPPC	Uncinate Process Pancreatic Cancers

Supplementary Table II: Quality assessment of included studies using JBI Critical Appraisal Checklist for Studies Reporting Prevalence Data.

Domains / Study	Was the sample representative of the target population?	Were study participants recruited in an appropriate way?	Was the sample size adequate?	Were the study subjects and setting described in detail?	Is the data analysis conducted with sufficient coverage of the identified sample?	Were objective, standard criteria used for measurement of the condition?	Was the condition measured reliably?	Was there appropriate statistical analysis?	Are all important confounding factors/ subgroups/differences identified and accounted for?	Were subpopulations identified using objective criteria?
**Alsabilah 2017**	Y	Y	Y	Y	Y	Y	Y	Y	Y	Y
**Kuzu 2017**	Y	Y	Y	Y	Y	Y	Y	Y	Y	Y
**Murono 2016**	Y	Y	Y	Y	Y	Y	Y	Y	Y	Y
**Gamo 2016**	Y	Y	Y	Y	Y	Y	Y	Y	Y	Y
**Haywood 2016**	N	U	N	Y	U	Y	Y	Y	U	U
**Lee 2016**	Y	Y	Y	Y	Y	Y	Y	Y	Y	Y
**Nesgaard 2015**	Y	Y	Y	Y	Y	Y	Y	Y	Y	Y
**Miyazawa 2015**	Y	Y	Y	Y	Y	Y	Y	Y	Y	Y
**Kaye 2015**	Y	Y	Y	Y	Y	Y	Y	Y	Y	Y
**Cao 2015**	Y	Y	Y	Y	Y	Y	Y	Y	Y	Y
**Ogino 2014**	Y	Y	Y	Y	Y	Y	Y	Y	Y	Y
**Spasojevic 2013**	N	Y	N	Y	Y	Y	Y	Y	U	U
**Hirai 2013**	Y	Y	Y	Y	Y	Y	Y	Y	Y	Y
**Tajima 2011**	Y	Y	Y	Y	Y	Y	Y	Y	Y	Y
**Spasojevic 2011**	N	Y	N	Y	Y	Y	Y	Y	Y	Y
**Sakaguchi 2010**	Y	Y	Y	Y	Y	Y	Y	Y	Y	Y
**Ignjatovic 2010**	N	N	N	Y	Y	Y	Y	Y	U	N
**Ignjatovic 2007**	N	N	N	Y	Y	Y	Y	Y	U	N
**Ferrari 2007**	Y	Y	Y	Y	Y	Y	Y	Y	Y	Y
**Jin 2006**	N	U	N	Y	Y	Y	Y	Y	U	U
**Cheng 2006**	Y	Y	Y	Y	Y	Y	Y	Y	Y	Y
**Ignjatovic 2004**	N	N	N	Y	Y	Y	Y	Y	U	N
**Shatari 2003**	N	U	N	Y	Y	Y	Y	Y	Y	Y
**Yamaguchi 2002**	N	Y	N	Y	Y	Y	Y	Y	U	U
**Yamada 2000**	N	U	N	Y	Y	Y	Y	Y	U	U

**Ito 2000**	N	Y	N	Y	Y	Y	Y	Y	U	U
**Lange 2000**	N	Y	N	Y	Y	Y	Y	Y	U	U
**Vedantham 1998**	N	U	N	Y	Y	Y	Y	Y	Y	Y
**Chung 1998**	Y	U	N	Y	Y	Y	Y	Y	U	U
**Yada 1997**	Y	Y	N	Y	Y	Y	Y	Y	Y	Y
**Graf 1997**	N	Y	N	Y	Y	Y	Y	Y	U	U
**Garcia-Ruiz 1996**	N	Y	Y	Y	Y	Y	Y	Y	U	U
**Zhang 1994**	Y	Y	Y	Y	Y	Y	Y	Y	Y	Y
**Crabo 1993**	N	Y	Y	Y	Y	Y	Y	Y	U	U
**Mori 1992**	N	U	N	Y	Y	Y	Y	Y	U	U
**VanDamme 1990**	U	Y	Y	Y	Y	Y	Y	Y	U	U
**Nelson 1988**	Y	Y	Y	Y	Y	Y	Y	Y	U	U
**Birtwisle 1983**	Y	Y	U	Y	Y	Y	Y	Y	U	U
**Michels 1965**	Y	Y	Y	Y	Y	Y	Y	Y	Y	Y
**Gillot 1964**	Y	Y	Y	Y	Y	Y	Y	Y	U	U
**Sonneland 1958**	Y	Y	Y	Y	Y	Y	Y	U	U	U
**Basmajian 1955**	U	Y	Y	Y	Y	Y	Y	Y	U	U
**Steward 1933**	Y	U	Y	U	U	U	Y	U	U	U
**Adachi 1928**	U	U	U	N	U	U	Y	U	U	U
**Jamieson 1909**	N	U	Y	U	U	U	Y	U	U	U
**Y- yes, N – No, U – unclear, NA – not applicable**										

Supplementary Table III: The subgroup analysis of the pooled prevalence of the superior mesenteric vein and artery anatomical variants related to colon and pancreatic resections.

Anatomical parameter	Subgroup of patients	Studies with more than 100 patients (Prevalence, 95%CI, I^2)	Studies from Europe (Prevalence, 95%CI, I^2)	Studies from Asia (Prevalence, 95%CI, I^2)	Studies from the USA (Prevalence, 95%CI, I^2)	Imagistic Studies (MDCT, Angiography) (Prevalence, 95%CI, I^2)	Surgical Studies (Prevalence, 95%CI, I^2)	Cadaveric studies (Prevalence, 95%CI, I^2)
**ICV presence**		0.998, 0.990-1.000, 0	-	0.997, 0.991-1.000, 0	-	-	0.997, 0.987-1.000, 0	0.997, 0.986-1.000, 0
**ICV drainage**	**SMV**	0.970, 0.873-1.000, 91.896	-	0.976, 0.941-1.000, 79.028	-	-	-	0.964, 0.874-1.000, 85.733
	**Henle trunk**	0.023, 0.000-0.120, 91.896	-	0.019, 0.000-0.056, 79.028	-	-	-	0.028, 0.000-0.120, 85.733
	**Jejunal trunk**	0.006, 0.000-0.072, 91.896	-	0.005, 0.000-0.028, 79.028	-	-	-	0.008, 0.000-0.070, 85.733
**ICA presence**		0.999, 0.997-1.000, 0.000	0.993, 0.985-0.999, 4.878	0.999, 0.997-1.000, 0.000	0.998, 0.994-1.000, 0.000	0.993, 0.980-1.000, 64.559	0.999, 0.996-1.000, 0.000	0.998, 0.994-1.000, 0.000
**ICA trajectory related to SMV**	**Anterior**	0.476, 0.415-0.537, 78.484	0.333, 0.262-0.407, 50.038	0.507, 0.458-0.556, 52.788	-	0.398, 0.276-0.526, 89.651	0.505, 0.400-0.610, 80.682	0.389, 0.313-0.469, 14.597
	**Posterior**	0.524, 0.462-0.584, 78.484	0.667, 0.591-0.736, 50.038	0.493, 0.444-0.542, 52.788	-	0.602, 0.472-0.723, 89.651	0.495, 0.390-0.600, 80.682	0.611, 0.531-0.687, 14.597
**RCV presence**		0.547, 0.201-0.865, 97.829	-	0.591, 0.364-0.801, 96.774	-	0.707, 0.371-0.960, 96.524	0.289, 0.078-0.555, 91.631	0.701, 0.270-1.000, 95.103
**RCV drainage**	**SMV**	0.627, 0.292-0.916, 96.113	-	0.490, 0.238-0.750, 95.608	-	0.418, 0.063-0.825, 96.872	-	0.353, 0.160-0.578, 70.961
	**ICV**	0.005, 0.000-0.110, 96.113	-	0.008, 0.000-0.087, 95.608	-	0.005, 0.000-0.144, 96.872	-	0.009, 0.000-0.075, 70.961
	**Henle trunk**	0.368, 0.084-0.708, 96.113	-	0.503, 0.250-0.762, 95.608	-	0.577, 0.175-0.937, 96.872	-	0.638, 0.422-0.840, 70.961
**RCVs number**	**One**	0.619, 0.000-1.000, 99.040	-	0.832, 0.000-1.000, 98.878	-	-	-	0.624, 0.000-1.000, 99.141
	**Two**	0.296, 0.000-1.000, 99.040	-	0.134, 0.000-1.000, 98.878	-	-	-	0.293, 0.000-1.000, 99.141
	**Three**	0.085, 0.000-1.000, 99.040	-	0.034, 0.000-1.000, 98.878	-	-	-	0.084, 0.000-1.000, 99.141
**SRCV presence**		0.741, 0.250-1.000, 98.592	-	0.682, 0.267-0.986, 98.187	-	0.715, 0.181-1.000, 98.627	-	0.568, 0.000-1.000, 92.306
**SRCV drainage**	**SMV**	0.063, 0.000-0.222, 92.709	-	0.046, 0.000-0.145, 86.457	-	0.042, 0.000-0.199, 92.924	-	0.031, 0.000-0.184, 87.957

	**RCV**	0.004, 0.000-0.074, 92.709	-	0.008, 0.000-0.061, 86.457	-	0.005, 0.000-0.091, 92.924	-	0.011, 0.000-0.127, 87.957
	**MCV**	0.004, 0.000-0.074, 92.709	-	0.015, 0.000-0.081, 86.457	-	0.005, 0.000-0.091, 92.924	-	0.031, 0.000-0.183, 87.957
	**Henle trunk**	0.930, 0.778-1.000, 92.709	-	0.0931, 0.828-1.000, 86.457	-	0.948, 0.801-1.000, 92.924	-	0.928, 0.502-1.000, 87.957
**RCA presence**		0.544, 0.335-0.745, 99.356	0.616, 0.245-0.931, 99.201	0.520, 0.330-0.707, 98.563	0.751, 0.472-0.956, 98.057	0.704, 0.315-0.983, 99.390	0.413, 0.131-0.726, 99.220	0.656, 0.461-0.828, 97.481
**RCA origin**	**SMA**	0.781, 0.287-0.656, 98.882	0.800, 0.407-0.807, 93.868	0.817, 0.253-0.681, 98.356	0.391, 0.165-0.497, 94.750	0.917, 0.485-0.948, 98.098	0.640, 0.144-0.651, 98.146	0.516, 0.170-0.485, 96.238
	**ICA**	0.121, 0.002-0.205, 98.882	0.093, 0.000-0.195, 93.868	0.114, 0.000-0.191, 98.356	0.242, 0.073-0.360, 94.750	0.043, 0.000-0.166, 98.098	0.178, 0.000-0.295, 98.146	0.221, 0.038-0.275, 96.238
	**MCA**	0.098, 0.000-0.184, 98.882	0.108, 0.000-0.239, 93.868	0.069, 0.000-0.145, 98.356	0.367, 0.150-0.477, 94.750	0.040, 0.000-0.161, 98.098	0.182, 0.000-0.299, 98.146	0.263, 0.054-0.308, 96.238
**RCA trajectory related to SMV**	**Anterior**	0.903, 0.865-0.929, 0.000	0.900, 0.822-0.944, 0.000	0.890, 0.827-0.941, 43.969	-	0.899, 0.857-0.929, 0.000	0.879, 0.774-0.955, 0.000	0.853, 0.659-0.979, 63.214
	**Posterior**	0.097, 0.068-0.131, 0.000	0.100, 0.047-0.165, 0.000	0.110, 0.059-0.173, 43.969	-	0.101, 0.068-0.138, 0.000	0.121, 0.045-0.226, 0.000	0.147, 0.021-0.341, 63.214
**ICA and RCA trajectory related to SMV**	**ICA anterior & RCA anterior**	0.186, 0.099-0.283, 89.449	-	0.160, 0.089-0.240, 84.521	-	0.169, 0.115-0.231, 56.028	-	-
	**ICA anterior & RCA posterior**	0.006, 0.000-0.031, 89.49	-	0.006, 0.000-0.028, 84.521	-	0.001, 0.000-0.009, 56.028	-	-
	**ICA anterior & RCA absent**	0.351, 0.235-0.460, 89.449	-	0.342, 0.243-0.438, 84.521	-	0.341, 0.269-0.414, 56.028	-	-
	**ICA posterior & RCA anterior**	0.100, 0.037-0.180, 89.449	-	0.109, 0.051-0.180, 84.521	-	0.139, 0.089-0.196, 56.028	-	-
	**ICA posterior& RCA posterior**	0.037, 0.003-0.097, 89.449	-	0.049, 0.012-0.103, 84.521	-	0.036, 0.012-0.071, 56.028	-	-
	**ICA posterior & RCA absent**	0.320, 0.209-0.428, 89.449	-	0.334, 0.235-0.429, 84.521	-	0.314, 0.244-0.386, 56.028	-	-
**MCV presence**		0.933, 0.802-1.000, 95.262	-	0.970, 0.912-1.000, 91.894	-	0.978, 0.930-1.000, 72.466	0.997, 0.987-1.000, 0.000	0.905, 0.722-1.000, 92.420
**MCVs number**	**One**	0.765, 0.589-0.909, 90.335	-	0.697, 0.514-0.858, 91.660	-	0.713, 0.243-1.000, 97.283	-	0.673, 0.363-0.927, 90.255
	**Two**	0.208, 0.074-0.383, 90.335	-	0.259, 0.111-0.441, 93.032	-	0.255, 0.000-0.722, 97.283	-	0.270, 0.042-0.581, 90.255
	**Three**	0.027, 0.000-0.105, 90.335	-	0.044, 0.000-0.137, 93.032	-	0.032, 0.000-0.314, 97.283	-	0.056, 0.000-0.237, 90.255
**MCV drainage**	**SMV**	0.870, 0.752-0.934, 84.865	-	0.832, 0.746-0.896, 80.303	-	0.769, 0.617-0.886, 86.025	-	0.849, 0.778-0.916, 21.470
	**Henle trunk**	0.094, 0.030-0.181, 84.865	-	0.117, 0.059-0.188, 80.303	-	0.171, 0.066-0.306, 86.025	-	0.084, 0.038-0.148, 21.470

	**SV**	0.012, 0.000-0.047, 84.865	-	0.015, 0.000-0.044. 80.303	-	0.006, 0.000-0.042, 86.025	-	0.017, 0.000-0.054, 21.470
	**IMV**	0.011, 0.000-0.046, 84.865	-	0.019, 0.000-0.050, 80.303	-	0.012, 0.000-0.059, 86.025	-	0.045, 0.012-0.095, 21.470
	**FJT**	0.013, 0.000-0.050, 84.865	-	0.018, 0.000-0.048, 80.303	-	0.041, 0.000-0.115, 86.025	-	0.005, 0.000-0.023, 21.470
**MCA presence**		0.974, 0.940-0.995, 87.192	0.916, 0.769-0.998, 95.271	0.983, 0.930, 1.000, 91.231	0.949, 0.903-0.982, 62.214	0.770, 0.425-0.993, 95.969	0.977, 0.934-1.000, 90.861	0.971, 0.936-0.993, 69.504
**MCA origin**	**SMA**	0.699, 0.000-1.000, 99.436	-	-	0.603, 0.000-1.000, 97.992	-	-	0.603, 0.000-1.000, 97.992
	**RCA**	0.295, 0.000-0.877, 99.436	-	-	0.318, 0.000-0.873, 97.992	-	-	0.318, 0.000-0.873, 97.992
	**HA**	0.001, 0.000-0.260, 99.436	-	-	0.012, 0.000-0.337, 97.992	-	-	0.012, 0.000-0.337, 97.992
	**SA**	0.001, 0.000-0.260, 99.436	-	-	0.003, 0.000-0.277, 97.992	-	-	0.003, 0.000-0.277, 97.992
	**LCA**	0.001, 0.000-0.260, 99.436	-	-	0.019, 0.000-0.369, 97.992	-	-	0.019, 0.000-0.369, 97.992
	**ICA**	0.001, 0.000-0.260, 99.436	-	-	0.019, 0.000-0.369, 97.992	-	-	0.019, 0.000-0.369, 97.992
	**Celiac artery**	0.001, 0.000-0.260, 99.436	-	-	0.012, 0.000-0.337, 97.992	-	-	0.012, 0.000-0.337, 97.992
	**IPDA**	0.001, 0.000-0.260, 99.436	-	-	0.012, 0.000-0.337, 97.992	-	-	0.012, 0.000-0.337, 97.992
**Henle trunk presence (RGEV + CVs +/- PVs)**		0.953, 0.876-0.997, 92.074	0.931, 0.811-1.000, 80.996	0.898, 0.812-0.961, 92.491	0.843, 0.609-1.000, 93.994	0.888, 0.762-0.974, 92.665	0.912, 0.809-0.980, 85.499	0.937, 0.832-1.000, 90.102
**Henle trunk types**	**GCT**	0.036, 0.000-1.000, 99.486	0.077, 0.000-0.334, 94.907	0.025, 0.000-0.302, 99.007	-	-	0.043, 0.000-0.545, 99.303	0.037, 0.000-0.166, 92.067
	**GPT**	0.281, 0.000-1.000, 99.486	0.378, 0.012-0.740, 94.907	0.312, 0.000-0.745, 99.007	-	-	0.465, 0.000-1.000, 99.303	0.256, 0.040-0.501, 92.067
	**GPCT**	0.668, 0.000-1.000, 99.486	0.535, 0.077-0.854, 94.907	0.649, 0.020-0.979, 99.007	-	-	0.474, 0.000-1.000, 99.303	0.697, 0.360-0.877, 92.067
	**CPT**	0.015, 0.000-1.000, 99.486	0.010, 0.000-0.169, 94.907	0.014, 0.000-0.265, 99.007	-	-	0.019, 0.000-0.470, 99.303	0.010, 0.000-0.098, 92.067
**Henle trunk forming veins**	**GCT (RGEV + SRCV)**	0.029, 0.000 – 0.193, 98.750	0.112, 0.000-0.661, 96.544	0.024, 0.000-0.138, 98.551	-	0.019, 0.000-0.213, 98.178	0.086, 0.000-0.389, 98.886	0.109, 0.000-0.259, 96.693
	**GPT (RGEV + ASPDV)**	0.205, 0.000-0.447, 98.750	0.416, 0.000-1.000, 96.544	0.225, 0.000-0.397, 98.551	-	0.106, 0.000-0.384, 98.178	0.434, 0.000-0.770, 98.886	0.323, 0.033-0.507, 98.886
	**PCT (ASPDV + SRCV)**	0.209, 0.000-0.137, 98.750	0.012, 0.000-0.379, 96.544	0.010, 0.000-0.105, 98.551	-	0.005, 0.000-0.160, 98.178	0.012, 0.000-0.221, 98.886	0.010, 0.000-0.093, 98.886

	**GPCT (RGEV + ASPDV + 1 Colic: RCV)**	0.042, 0.000-0.219, 98.750	0.012, 0.000-0.379, 96.544	0.091, 0.000-0.246, 98.551	-	0.072, 0.000-0.329, 98.178	0.023, 0.000-0.255, 98.886	0.046, 0.000-0.168, 98.886
	**GPCT (RGEV + ASPDV + 1 Colic: SRCV)**	0.519, 0.048-0.744, 98.750	0.333, 0.000-1.000, 96.544	0.344, 0.019-0.533, 98.551	-	0.471, 0.000-0.759, 98.178	0.358, 0.000-0.707, 98.886	0.306, 0.027-0.492, 98.886
	**GPCT (RGEV + ASPDV + 1 Colic: MCV)**	0.010, 0.000-0.142, 98.750	0.033, 0.000-0.470, 96.544	0.018, 0.000-0.126, 98.551	-	0.016, 0.000-0.204, 98.178	0.010, 0.000-0.212, 98.886	0.022, 0.000-0.125, 98.886
	**GPCT (RGEV + ASPDV + 1 Colic: ICV)**	0.007, 0.000-0.127, 98.750	0.012, 0.000-0.379, 96.544	0,008, 0.000-0.100, 98.551	-	0.005, 0.000-0.160, 98.178	0.005, 0.000-0.186, 98.886	0.14, 0.000-0.104, 98.886
	**GPCT (RGEV + ASPDV + 2 Colic: RCV + SRCV)**	0.094, 0.000-0.308, 98.750	0.012, 0.000-0.379, 96.544	0.158, 0.000-0.327, 98.551	-	0.126, 0.000-0.414, 98.178	0.044, 0.000-0.309, 98.886	0.091, 0.000-0.236, 98.886
	**GPCT (RGEV + ASPDV + 2 Colic: RCV + MCV)**	0.008, 0.000-0.134, 98.750	0.012, 0.000-0.379, 96.544	0.034, 0.000-0.159, 98.551	-	0.048, 0.000-0.285, 98.178	0.010, 0.000-0.212, 98.886	0.016, 0.000-0.110, 98.886
	**GPCT (RGEV + ASPDV + 2 Colic: SRCV + MCV)**	0.039, 0.000-0.215, 98.750	0.012, 0.000-0.379, 96.544	0.030, 0.000-0.151, 98.551	-	0.063, 0.000-0.314, 98.178	0.005, 0.000-0.186, 98.886	0.013, 0.000-0.103, 98.886
	**GPCT (RGEV + ASPDV + 2 Colic: RCV + ICV)**	0.007, 0.000-0.131, 98.750	0.012, 0.000-0.379, 96.544	0.009, 0.000-0.102, 98.551	-	0.005, 0.000-0.160, 98.178	0.005, 0.000-0.186, 98.886	0.015, 0.000-0.107, 98.886
	**GPCT (RGEV + ASPDV + 3 Colic: RCV + SRCV + MCV)**	0.024, 0.000-0.180, 98.750	0.012, 0.000-0.379, 96.544	0.037, 0.000-0.163, 98.551	-	0.052, 0.000-0.291, 98.178	0.005, 0.000-0.186, 98.886	0.023, 0.000-0.125, 98.886
	**GPCT (RGEV + ASPDV + 3 Colic: RCV + SRCV + ICV)**	0.007, 0.000-0.127, 98.750	0.012, 0.000-0.379, 96.544	0.012, 0.000-0.109, 98.551	-	0.011, 0.000-0.188, 98.178	0.005, 0.000-0.186, 98.886	0.014, 0.000-0.104, 98.886

CI – confidence interval; CPT – colo-pancreatic trunk; GCT – gastro-colic trunk; GPT – gastro-pancreatic trunk; GPCT gastro-pancreato-colic trunk; HA – hepatic artery; ICA – ileocolic artery; ICV – ileocolic vein, IMV – inferior mesenteric vein; IPDA – inferior pancreaticoduodenal artery; FJT – first jejunal trunk; LCA – left colic artery; MCA – middle colic artery; MCV – middle colic vein; ASPDV – anterosuperior pancreaticoduodenal vein; RGEV – right gastroepiploic vein; RCA – right colic artery; RCV – right colic vein; SMA – superior mesenteric artery; SMV – superior mesenteric vein; SV – splenic vein; SA – splenic artery; SRCV – superior right colic vein.

Supplementary Table IV: Sensitivity analysis undertaking one study removed analysis revealing no relevant changes in the overall effects of the quantitative synthesis for the pooled prevalence of the Henle trunk presence.

Excluded study	Pooled Prevalence	LCI 95%	HCI 95%	Cochran Q	p	I 2	I 2 LCI 95%	I 2 HCI 95%
Alsabilah 2017	0.902484	0.834632	0.954568	201.3081	0	91.55523	88.15604	93.97886
Kuzu 2017	0.885248	0.818307	0.938851	171.7306	0	90.10077	85.88959	93.05515
Lee 2016	0.892939	0.821029	0.948746	203.3904	0	91.64169	88.28936	94.03437
Cao 2015	0.894665	0.82174	0.950853	206.3423	0	91.76126	88.47346	94.11126
Miyazawa 2015	0.885538	0.817992	0.939499	176.3335	0	90.35918	86.29542	93.21793
Ogino 2014	0.898456	0.827929	0.952688	208.5219	0	91.84738	88.60586	94.16672
Ignjatovic 2010	0.901327	0.833291	0.953679	205.8569	0	91.74184	88.44357	94.09876
Sakaguchi 2010	0.903046	0.83543	0.954913	195.6853	0	91.31258	87.781	93.82345
Jin 2006	0.892792	0.824043	0.946678	208.105	0	91.83105	88.58076	94.15619
Ignjatovic 2004	0.892439	0.823563	0.946445	207.8871	0	91.82248	88.5676	94.15068
Yamaguchi 2002	0.905786	0.841005	0.95559	191.2477	0	91.11101	87.46849	93.69476
Lange 2000	0.89765	0.828351	0.951253	209.0164	0	91.86667	88.63549	94.17915
Ito 2000	0.896193	0.826544	0.9502	209.0835	0	91.86928	88.6395	94.18083
Vedantham 1998	0.891858	0.821665	0.946751	205.7327	0	91.73685	88.4359	94.09555
Zhang_cadaveric 1994	0.894313	0.823812	0.949083	208.0176	0	91.82761	88.57549	94.15398
Zhang_CT 1994	0.91007	0.850296	0.956309	171.7089	0	90.09952	85.88762	93.05436
Crabo 1993	0.89777	0.826399	0.9526	208.9529	0	91.86419	88.63169	94.17756
Birtwisle 1983	0.887343	0.817836	0.942351	195.5734	0	91.30761	87.7733	93.82027
Gillot 1964	0.908539	0.848729	0.954992	166.0021	0	89.75916	85.35109	92.84078

Supplementary Table V: The search strategy used in PubMed/Medline database.

Search	Query	Items found
#7	Search (((((superior mesenteric vein[Title/Abstract]) OR superior mesenteric artery[Title/Abstract]) OR portal vein[Title/Abstract]) OR gastrocolic trunk[Title/Abstract])) AND (((((anatomy[Title/Abstract]) OR anatomical variation[Title/Abstract]) OR variations[Title/Abstract]) OR variant[Title/Abstract]) OR variability[Title/Abstract])	1416
#6	Search ((((anatomy[Title/Abstract]) OR anatomical variation[Title/Abstract]) OR variations[Title/Abstract]) OR variant[Title/Abstract]) OR variability[Title/Abstract]	663226
#5	Search (((superior mesenteric vein[Title/Abstract]) OR superior mesenteric artery[Title/Abstract]) OR portal vein[Title/Abstract]) OR gastrocolic trunk[Title/Abstract]	30592
#4	Search gastrocolic trunk[Title/Abstract]	49
#3	Search portal vein[Title/Abstract]	23167
#2	Search superior mesenteric artery[Title/Abstract]	7223
#1	Search superior mesenteric vein[Title/Abstract]	1555
